# Author Correction: Immune cell profiling of COVID-19 patients in the recovery stage by single-cell sequencing

**DOI:** 10.1038/s41421-020-00187-5

**Published:** 2020-06-20

**Authors:** Wen Wen, Wenru Su, Hao Tang, Wenqing Le, Xiaopeng Zhang, Yingfeng Zheng, Xiuxing Liu, Lihui Xie, Jianmin Li, Jinguo Ye, Liwei Dong, Xiuliang Cui, Yushan Miao, Depeng Wang, Jiantao Dong, Chuanle Xiao, Wei Chen, Hongyang Wang

**Affiliations:** 1 National Center for Liver Cancer, Second Military Medical University, 200438 Shanghai, China; 2 State Key Laboratory of Ophthalmology, Zhongshan Ophthalmic Center, Sun Yat-sen University, 510060 Guangzhou, China; 3 Department of Respiratory and Critical Care Medicine, Changzheng Hospital, Second Military Medical University, 200003 Shanghai, China; 4 Department of Critical Care, Wuhan Huoshenshan Hospital, 430113 Wuhan, Hubei China; 5 Department of Critical Care, Wuhan Hankou Hospital, 430000 Wuhan, Hubei China; 6 Laboratory of Vaccine and Antibody Engineering, Beijing Institute of Biotechnology, 100071 Beijing, China; 7 GrandOmics Diagnosis Co. Ltd., 430014 Wuhan, Hubei China; 8 Berry Genomics Co. Ltd., 102206 Beijing, China; 9 Eastern Hepatobiliary Surgery Hospital, Second Military Medical University, 200438 Shanghai, China; 10 Ministry of Education (MOE) Key Laboratory of Signaling Regulation and Targeting Therapy of Liver Cancer, Second Military Medical University, 200433 Shanghai, China

Correction to: *Cell Discovery* (2020) 6:31

10.1038/s41421-020-0168-9 Published online 04 May 2020

Following publication of the original article^1^, an error was identified in the Data availability section. The accession number of the dataset was updated. The corrected Data availability appears below:

The raw sequence data reported in this paper has been deposited in the Genome Sequence Archive (Genomics, Proteomics & Bioinformatics 2017) in National Genomics Data Center (Nucleic Acids Res 2020), Beijing Institute of Genomics (China National Center for Bioinformation), Chinese Academy of Sciences, under Project Accession No. PRJCA002413 (GSA Accession No. CRA002497) that are publicly accessible at https://bigd.big.ac.cn/gsa.
