# Supplementary material for: Immune cell profiling of COVID-19 patients in the recovery stage by single-cell sequencing
Source: Cell Discov. 2020 May 4;6:31. doi: 10.1038/s41421-020-0168-9 (PMC7197635; doi:10.1038/s41421-020-0168-9)
Supplement: Supplementary file 1 — Supplementary Information [file 41421_2020_168_MOESM1_ESM.pdf]

a

a: clinical data of the enrolled subjects: ERS and LRS	ERS-1	ERS-2	ERS-3	ERS-4	ERS-5	LRS-1	LRS-2	LRS-3	LRS-4	LRS-5
**Gender**	male	male	female	female	female	male	male	male	female	female
**Smoking History**	-	now	-	-	-	once	now	now	now	-
**Wuhan Exposure History**	+	+	+	+	+	+	+	+	+	+
**First Negative Nucleic Acid Transfer**	February 16, 2020	February 19, 2020	February 7, 2020	February 14, 2020	February 8, 2020	February 3, 2020	February 8, 2020	February 13, 2020	January 29, 2020	February 1, 2020
**Severity**	moderate	moderate	severe	severe	moderate	severe	moderate	moderate	severe	severe
**Fever**	+	+	+	+	+	+	+	+	+	+
**Conjunctival Congestion**	-	-	-	-	-	-	-	-	-	-
**Rhinobyon**	-	-	-	-	-	-	+	-	-	-
**Cough**	+	+	-	+	+	+	-	+	+	+
**Sore throat**	+	+	-	+	+	+	-	+	+	+
**Expectoration**	+	+	+	+	+	+	-	+	+	+
**Exhaustion**	+	+	+	-	+	+	+	+	+	+
**Hemoptysis**	-	+	-	+	-	-	-	-	+	-
**Breathe Hard**	-	+	+	+	+	+	+	+	+	+
**Emesis**	-	+	-	-	-	-	+	-	+	-
**Diarrhea**	-	-	-	-	-	-	+	-	-	-
**Muscle or Joint Pain**	-	+	-	+	+	+	+	+	+	+
**Chilly**	+	+	-	+	+	+	+	+	+	+
**Throat Congestion**	+	+	+	+	+	+	-	-	+	+
**Tonsil Edema**	-	+	+	+	-	-	-	-	-	+
**Lymphadenovarix**	-	-	-	-	-	-	-	-	-	-
**Rash**	-		-	-	-	-	-	-	-	-
**CT Finding**	Bilaeral pneumonia	Bilaeral pneumonia	Bilaeral pneumonia	Bilaeral pneumonia	Bilaeral pneumonia	Bilaeral pneumonia	Bilaeral pneumonia	Bilaeral pneumonia	Bilaeral pneumonia	Bilaeral pneumonia
**Chronic Basic Disease**	coronary heart disease	tumor and gastritis	HBV	diabetes	hypertension	hypertension; coronary heart disease	hypertension; coronary heart disease; HBV	hypertension	hypertension; coronary heart disease; diabetes	-
**Ventilator**	-	-	-	+	-	-	-	-	-	-
**Methylprednisolone**	-	-	40mg	80mg	40mg	40mg	-	40mg	40mg	80mg
**Antiviral Drug**	Oseltamivir	Lopinavir and Ritonavir Tablets;Oseltamivir	Oseltamivir	Ritonavir; Oseltamivir	Oseltamivir	Oseltamivir	Lopinavir and Ritonavir Tablets; Oseltamivir	Oseltamivir	Oseltamivir	Lopinavir and Ritonavir Tablets; Oseltamivir

b

b: clinical data of the enrolled subjects: HC	HC-1	HC-2	HC-3	HC-4	HC-5
**Gender**	male	male	female	female	female
**Chronic Basic Disease**	-	-	-	-	-

c

c	HC				ERS				LRS				
**variable**	mean	SD	median	percent	mean	SD	median	percent	mean	SD	median	percent	P value
**Age (years)**	55.4	14.79	55		58	11.77	58		55.2	17.46	49		0.78
**Gender (male, %)**				40				40				60	0.76

a

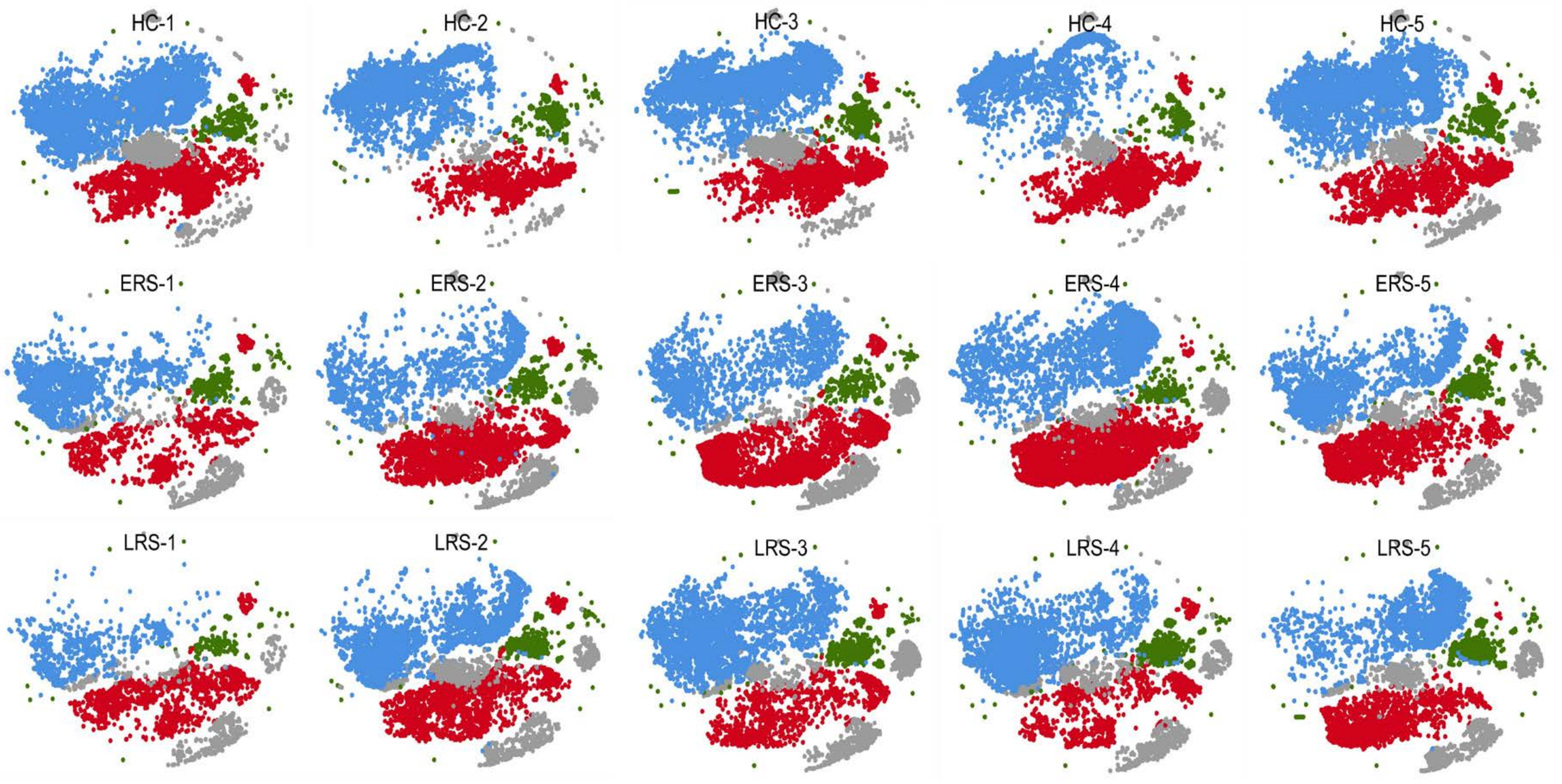
HC-1
HC-2
HC-3
HC-4
HC-5
ERS-1
ERS-2
ERS-3
ERS-4
ERS-5
LRS-1
LRS-2
LRS-3
LRS-4
LRS-5

Supplementary Fig. S3

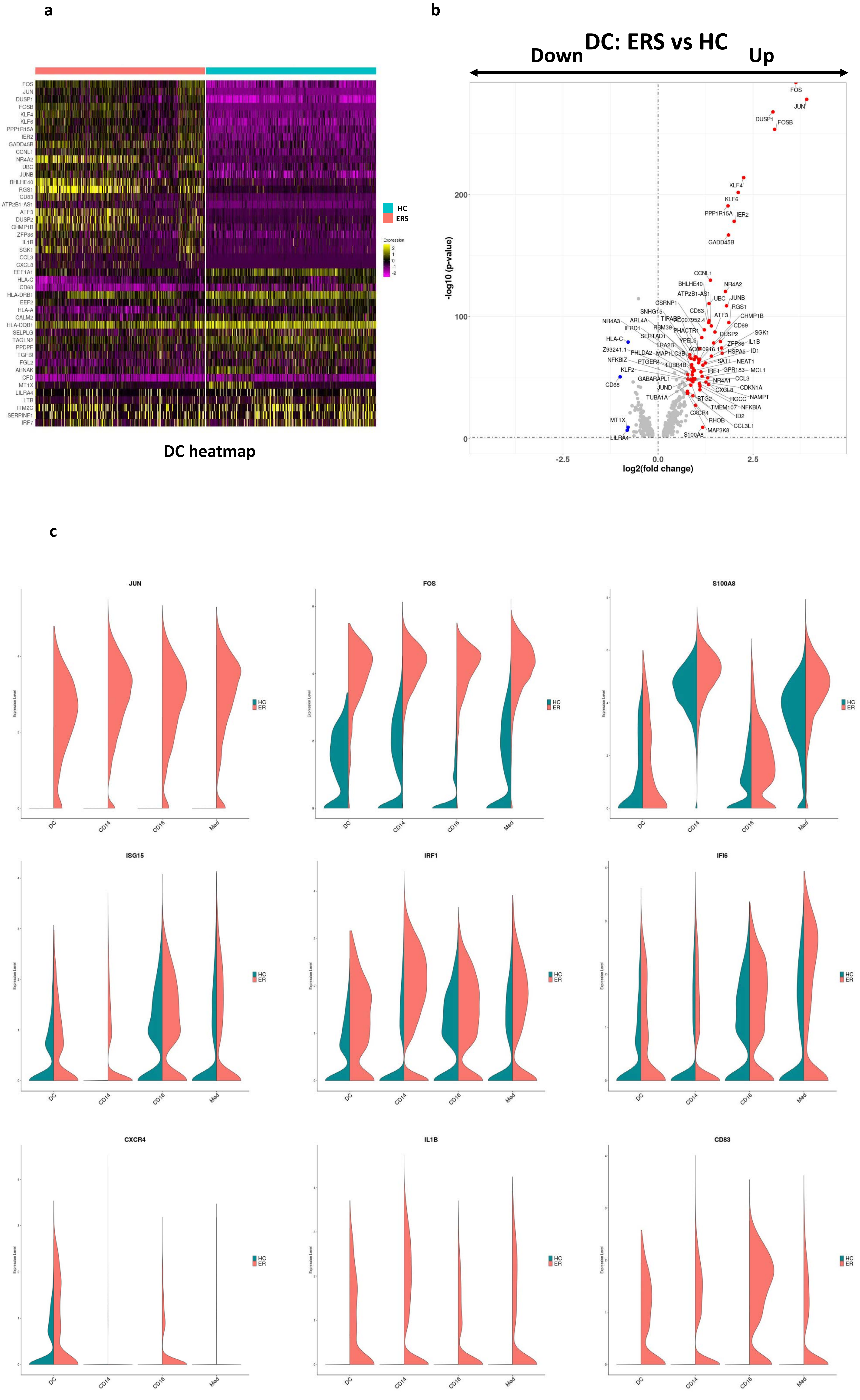

Supplementary Fig. S4

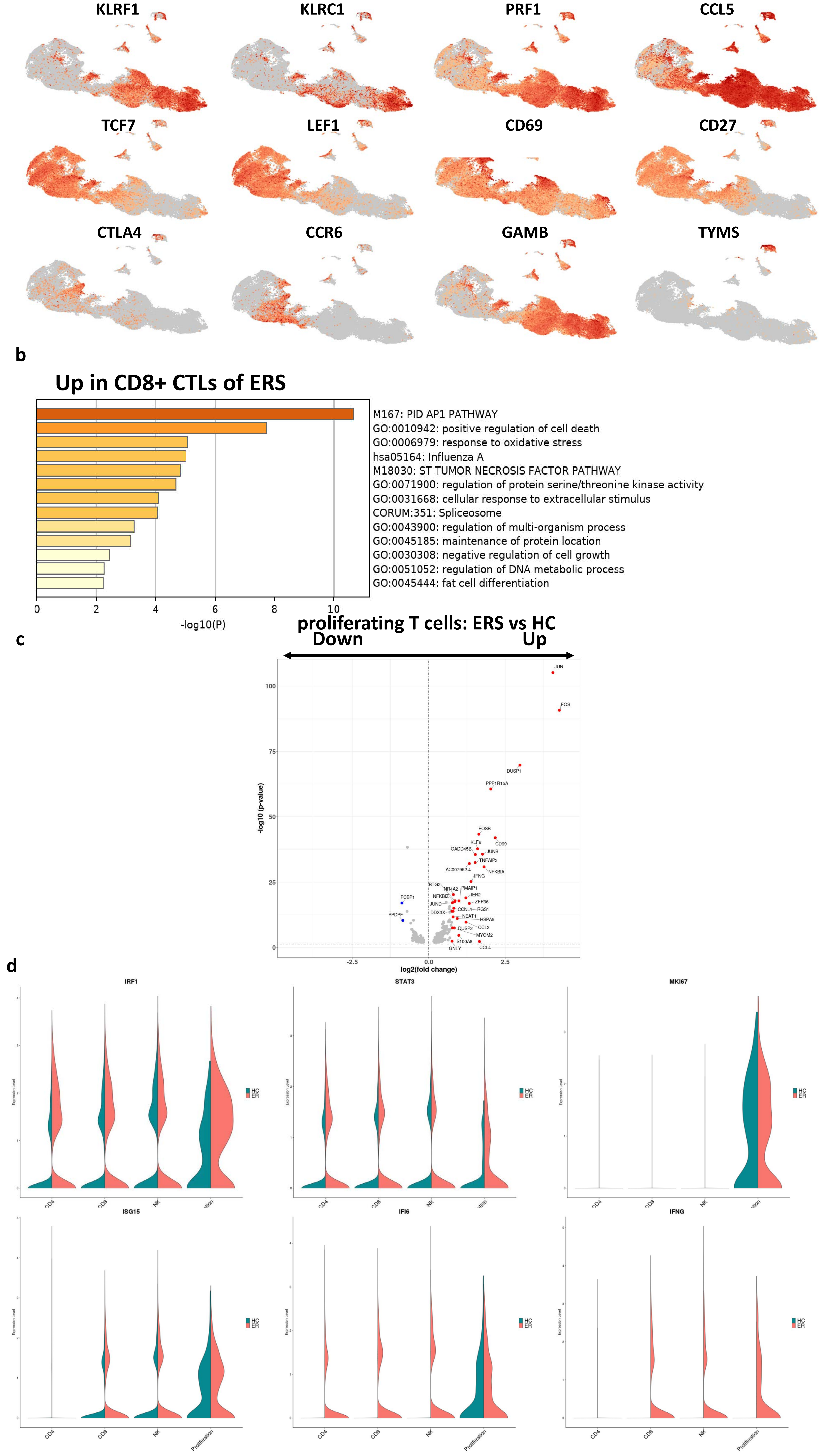

Supplementary Fig. S5

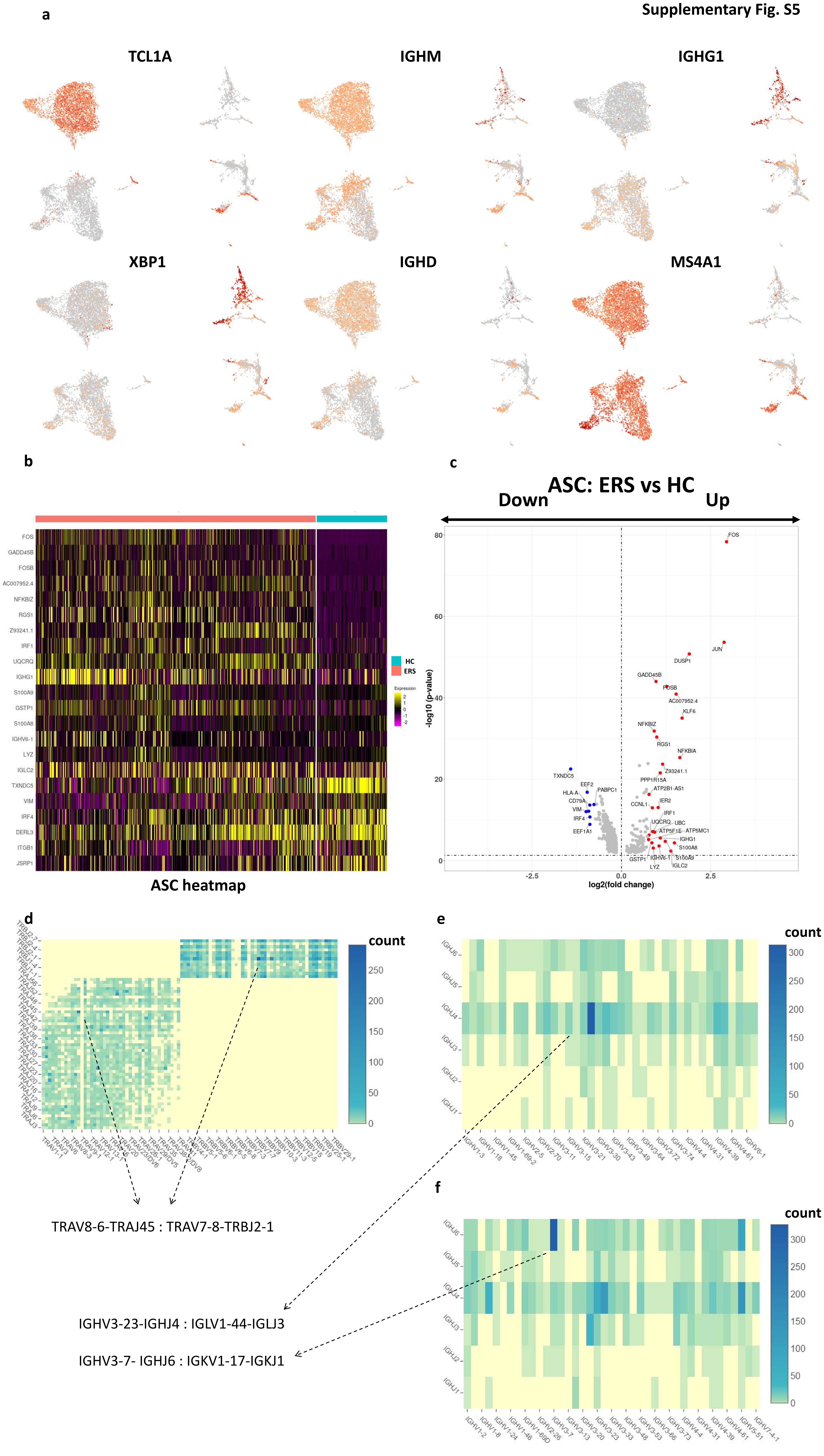

Figure legends

Supplementary figure S1. The detail information of patients and healthy controls.

(a-b) The clinical characteristics of the patients (a) and healthy donors (b) are reported in Supplementary figure S1.

(c) No significant differences were detected between HC, ERS group and LRS group. P-Value for categories were calculated using Chi-square analysis. P-value for numerical values were determined using One-Way ANOVA test.

Supplementary figure S2.

(a) tSNE plot showing the myeloid cells (red), NK&T cells (blue), B cells (green) and other clusters (grey) of PBMCs of each research object.

Supplementary figure S3. Analysis of myeloid cells subsets landscape in COVID-19 patients.

(a) The heatmap of DCs showing the DEGs between COVID-19 patients and HCs.

(b) The volcano plot shows the DEGs of DCs between COVID-19 patients and HCs.

(c) The violin plot shows the *JUNB, FOS, S100A8, ISG15, IRF1, IFI6, CXCR4, IL1β, CD83* were highly expressed in COVID-19 patients vs HCs in myeloid cells. P values were calculated using a paired, two-sided Wilcoxon test and FDR corrected using the Benjamini–Hochberg procedure.

Supplementary figure S4. Analysis of NK and T subsets landscape in COVID-19 patients.

(a) The UAMP plot showing subtype-specific marker genes of NK and T cells including *KLRF1, KLRC1, PRF1, CCL5, TCF7, LEF1, CD69, CD27, CTLA4, CCR6, GZMB and TYMS*.

(b) GO BP enrichment analysis of the DEGs of $CD8^+$ CTLs between the COVID-19 patients vs. the HCs. P value was derived by a hypergeometric test.

(c) The volcano plot shows the DEGs of ERS COVID-19 patients vs. HCs in proliferating T cells. P values were calculated using a paired, two-sided Wilcoxon test and FDR corrected using the Benjamini–Hochberg procedure.

(d) The violin plot shows the *IRF1, STAT3, MKI67, ISG15, IFI6 and IFNG* were highly expressed in ERS COVID-19 patients vs HCs in NK and T sub-clusters.

Supplementary figure S5. Analysis of B cell subsets landscape in COVID-19 patients.

(a) The UAMP plot showing subtype-specific marker genes of B cells including TCL1A, IGHM, IGHG1, XBP1, IGHD and MS4A1.

(b) The heatmap of ASCs showing the DEGs between COVID-19 patients and HCs.

(c) The volcano plot shows the DEGs of COVID-19 patients vs. HCs in ASCs. P values were calculated using a paired, two-sided Wilcoxon test and FDR corrected using the Benjamini–Hochberg procedure.

(d) Heat map showing TRA and TRB rearrangements in peripheral blood samples from ERS group.

(e) Heat map showing IGH rearrangements in peripheral blood samples from ERS-4 sample.

(f) Heat map showing IGH rearrangements in peripheral blood samples from ERS-5 sample.
